# Supplementary material for: Organic–Inorganic Hybrid Pigments Based on Bentonite: Strategies to Stabilize the Quinoidal Base Form of Anthocyanin
Source: Int J Mol Sci. 2023 Jan 26;24(3):2417. doi: 10.3390/ijms24032417 (PMC9917136; doi:10.3390/ijms24032417)
Supplement: Supplementary file 1 [file ijms-24-02417-s001.zip › ijms-2050280-supplementary.pdf]

Supplementary Material

In the CHN analysis, the cystine standard was made to confirm that the equipment is calibrated, the Bent bentonite clay sample before the ammonium acetate (AmAC) process and the clay after the Bent-AmAC ammonium acetate process as described in section 3.2. The CEC result of the bentonite clay was 75 cmol(+)/kg, as shown in Table S1.

Table S1. CNH analysis of Bent clays without ammonium acetate and Bent-AmAC with ammonium acetate.

Run Details		Results				CEC	Average CEC	CEC standard deviation
**Run**	**Weight**	**Carbon**	**Hydrogen**	**Nitrogen**	**mmol de N/ g of clay**	**cmol(+)/kg**	**cmol(+)/kg**	**cmol(+)/kg**
C	1.493	29.86	4.71	11.58				
C	1.695	30.16	5.27	11.7				
Bent	1.595	0.11	1.14	-				
Bent	1.835	0.33	0.96	-				
Bent	1.478	0.37	1	-				
Bent-AmAC	1.923	1.06	1.34	1.03	0.736	73.571		
Bent-AmAC	2.237	1.02	1.34	1.08	0.771	77.143	75.238	1.798
Bent-AmAC	1.944	1.04	1.34	1.05	0.750	75.000		

C corresponds to the cystine pattern. Reference values are: C 29.99% H 5.03% N 11.67% and S 26.69%
